# Correlation between T_H_1 response standard cytokines as biomarkers in patients with the delta virus in the western Brazilian Amazon

**DOI:** 10.1590/0074-02760160035

**Published:** 2016-04

**Authors:** Larissa Deadame de Figueiredo Nicolete, Lourdes Maria Pinheiro Borzacov, Deusilene Souza Vieira, Roberto Nicolete, Juan Miguel Villalobos Salcedo

**Affiliations:** 1 Universidade Federal de Rondônia, Programa de Pós-Graduação em Biologia Experimental, Porto Velho, RO, Brasil; 2 Centro de Pesquisa em Medicina Tropical, Porto Velho, RO, Brasil; 3 Fundação Oswaldo Cruz, Laboratório de Biotecnologia Aplicada à Saúde, Porto Velho, RO, Brasil

**Keywords:** HDV, T_H_1 cytokines, patients treated

## Abstract

Hepatitis D virus (HDV) is endemic in the Amazon Region and its pathophysiology is the most severe among viral hepatitis. Treatment is performed with pegylated interferon and the immune response appears to be important for infection control. HDV patients were studied: untreated and polymerase chain reaction (PCR) positive (n = 9), anti-HDV positive and PCR negative (n = 8), and responders to treatment (n = 12). The cytokines, interleukin (IL)-2 (p = 0.0008) and IL-12 (p = 0.02) were differentially expressed among the groups and were also correlated (p = 0.0143). Future studies will be conducted with patients at different stages of treatment, associating the viral load with serum cytokines produced, thereby attempting to establish a prognostic indicator of the infection.

Hepatitis delta is considered the most severe form of viral hepatitis and is caused by the hepatitis D virus (HDV) (Pascarella & Negro 2011). This virus is a RNA, hepatotropic virus, and is dependent on the hepatitis B virus (HBV), since HDV uses the HBV surface antigen (HBsAg) for the assembly of new viral particles (Karayiannis 1998). Currently, there are 240 million people positive for HBsAg worldwide (Ott et al. 2012), making the prevalence of HDV infection about 15 million carriers. In Brazil, the endemic areas correspond to states of the western Amazon Region (Acre, Amazonas, Rondônia, and Roraima) with a prevalence of 41.9% among carriers of HBsAg (Braga et al. 2012).

The most recent studied treatments consist of the association between pegylated interferon (PEG-IFN) and HBV reverse transcriptase inhibitors, as adefovir and tenofovir, for extremely long periods (Heidrich et al. 2013, 2014, Lunemann et al. 2015).

In order to reinforce the importance of the host immune response against viral infection, this study investigated whether serum cytokines could indicate some response in the antiviral therapy of patients who achieved a negative HDV RNA at week 24, consistent with a virological response against HDV. Therefore, the cytokines interleukin (IL)-2, IL-10, IL-12, IFN-γ, and tumour necrosis factor-alpha were quantified using the ELISA method (Opteia, USA). Nine untreated patients and polymerase chain reaction (PCR) positive for HDV RNA (HDV positive), eight anti-HDV positive and PCR negative for HDV patients (HDV negative), 12 patients with HDV who ended the specific antiviral treatment remained PCR RNA negative for the virus six months after the treatment protocol ended (HDV TTO) (Ethical Committee approval: 146.474 of 11/14/2012 CAAE 08485112.4.0000.5300).

After the quantification of cytokines in the patients’ serum, the Kruskal-Wallis test was used followed by Dunn’s post-test in order to compare the results obtained. A p-value < 0.05 was considered significant. Among all the cytokines tested, IL-2 and IL-12 were shown to be differentially expressed with values of p = 0.0008 (A in Figure) and p = 0.02 (B in Figure), respectively. The increase in IL-2 and IL-12 showed a significant positive correlation (p = 0.0143) after Spearman analysis (C in Figure).

**Figure:**
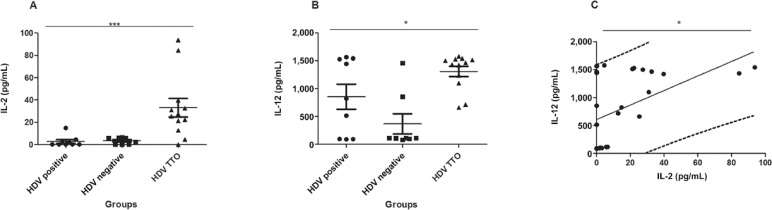
A: the significant difference of the interleukin (IL)-2 cytokine (p = 0.0008) in patients who completed treatment. The statistical tests used were the Krusal-Wallis test followed by the Dunn post-test; B: the same statistical test for the cytokine IL-12. The p-value was significant (p = 0.002); C: Spearman analysis. The values of IL-2 vs. IL-12 were positively correlated (p = 0.0143); HDV: hepatitis D virus.

One study analysed the profile of cytokines in HDV patients during treatment with PEG-IFN and associated the virological response of subjects who were responders with those who produced IL-2, IFN-γ, and inducible protein-10 (Grabowski & Wedemeyer 2010). Our results also showed that the production of IFN-γ by patients presented medians of 0.69, 2.77, and 1.27 pg/mL (data not shown) in the groups HDV positive, HDV negative, and HDV TTO, respectively, suggesting a decrease in production in the groups in which HDV is replicating.

With respect to IL-2, the same above mentioned authors suggest that, despite the effects of treatment with PEG-IFN, patients who responded and who present decreased HDV viral load must have an antigen-specific T-cell dependent cellular immune response. Our study also suggested that the exacerbation of IL-2 is not observed in the other groups and is important in the virological response after the end of treatment. Perhaps this is so because of the importance of this cytokine in the clonal expansion of specific cells that fights the virus effectively (Nijkamp & Parnham 2011, Boyman & Sprent 2012).

Patients who responded to treatment also presented an elevated quantification of IL-12. Although it is usually not a cytokine analysed in patients with HDV, some authors have correlated the increase of this cytokine in HBV patients, when treatment of this disease was performed with IFN-α (Cavanaugh et al. 1997,Ozkan et al. 2010). Our results suggest that IL-12 may be important in those patients in whom the HDV virus is replicating represented by the HDV positive group.

By analysing the correlation obtained between IL-12 and IL-2, the polarisation of a T_H_1 standard cell response is strongly suggested in patients who completed treatment (Boyman & Sprent 2012). Although there are no reports with HDV, the participation of peripheral cells in HBV infection is well established (Jung & Pape 2002). Human leukocyte antigen class II restricted CD4^+^ helper T-cells are responsible for the recognition of innumerable viral antigens and also for the mechanisms which lead to vaccine protection against the virus (Caillat-Zucman et al. 1998). Thus, our results support the T-cell homing hypothesis (Grabowski et al. 2011) in order for individuals to remain HDV RNA negative after the end of the treatment protocol, since the HBV envelope is essential for delta virus replication and can support assembly and release of new infectious HDV particles (Freitas et al. 2014). Thus, the results obtained in this study suggest that IL-2 is a potential biomarker for the indication of a good prognosis for the patient. Regarding the importance of these cytokines for the maintenance of an effective viral response, we believe that the different levels of the produced cytokines measured may occur due to the dynamics of the treatment protocol. Future studies with IL-2, IL-12, and IFN-γ will be conducted with patients at different stages of treatment in order to better understand how the response to treatment may possibly be dependent on the status of the host immune system. Therefore, its cellular and humoral parameters should be further studied and taken into account when improving the current treatment protocol.
